# Probability and pattern of occult cervical lymph node metastases in primary parotid carcinoma

**DOI:** 10.1007/s00405-016-4407-5

**Published:** 2016-11-28

**Authors:** Dominik Stodulski, Bogusław Mikaszewski, Hanna Majewska, Piotr Wiśniewski, Czesław Stankiewicz

**Affiliations:** 1 Department of Otolaryngology, Medical University of Gdańsk, Gdańsk, Poland; 2 Department of Pathomorphology, Medical University of Gdańsk, Gdańsk, Poland; 3 Department of Endocrinology and Internal Medicine, Medical University of Gdańsk, Gdańsk, Poland

**Keywords:** Parotid carcinoma, Occult, Lymph node metastases, Lymph node groups, Cervical, Regional, Elective neck dissection

## Abstract

The present study was undertaken to evaluate real probability and pattern of cervical occult lymph node metastases (OLNM) in primary parotid carcinoma (PPC). We carried out a retrospective analysis of 66 patients treated in years 1992–2010 due to PPC, who underwent elective neck dissection (END). In search of risk factors for OLNM, we analysed the following parameters: age, sex, pT-Status, tumour size, skin invasion, facial nerve palsy, tumour fixation, extraparotid extension, localization, grade, histology, intra/periparotid LN metastases (IPLNM). OLNM was observed in 30.3% of patients. In a univariate analysis statistical significance was found for IPLNM, extraparotid extension and high risk histology. A multivariate analysis showed statistical significance only for the first variable. The most common location of cervical OLNM was level II (80%), then III (45%) and V (30%). In a compilation of our own material with data from the literature (5 series), we obtained a group of 80 patients with OLNM, selected out of 650 patients with cN0 (12.3%). The proportion of metastases to particular levels was the following: 69%—II, 22.5%—III, 20%—I,16%—V, 7.5%—IV. END should be carried out in case of all T3/T4a carcinomas with minimal range of levels II and III. Removal of levels Ib and Va is recommended as well. In the T1/T2 carcinomas with high grade/high risk histology, END should be performed including levels II and III.

## Introduction

Primary parotid carcinoma (PPC) is a relatively rare (0.3% of all malignancies) and histologically diverse disease [1]. Therefore, data on this neoplasm are usually based on reports pertaining to groups of patients from single institutions, amounting to approximately 100 cases over many years. This is also associated with the problem of metastases to cervical lymph nodes, whose presence significantly increases the risk of locoregional relapse and death, worsening 5- and 10-year OS and DFS by approximately 50% [2–5]. Metastases in N0 nodes (occult) constitute an exceptional problem. This is because in many cases resigning from elective neck dissection (END) in fear of overtreatment can decrease the chances for recovery. Evaluation of actual prevalence and location of occult metastases in PPC based in the literature is made difficult due to frequent presentation of all major salivary gland tumours and mixing intra/periparotid metastases with neck metastases. Accordingly, the authors analysed their own material and data from the worldwide literature to identify the probability and probable locations of cervical occult lymph node metastases in PPC.

## Patients and methods

The study was conducted according to the Declaration of Helsinki on biomedical research involving human subjects. In years 1992–2010, in the Department of Otolaryngology, there were 111 patients treated due to primary parotid gland carcinoma. In 66 patients (59%), there were no enlarged lymph nodes (cN0) diagnosed based on clinical examination and imaging examinations (ultrasound, CT or MRI performed in 100, 35 and 28%, respectively). Clinical and pathological data of 66 patients who underwent elective neck dissection (END) was analysed, retrospectively. The investigated group comprised 34 women (51.5%) and 32 men (48.5%), aged 11 to 90 years (mean age 60.4 years). Local stage assessed based on TNM staging system from year 2009 was the following: T1–5 (7.6%), T2–23 (34.8%), T3–9 (13.6%), and T4a–29 (43.9%) [6]. In 27 patients parotidectomy with nerve VII preservation (partial −19, total −8), 13 patients underwent total parotidectomy with preservation of only part of the nerve (semiconservative), and in the remaining 26 cases radical parotidectomy was carried out (including parotidectomy with removal of the surrounding structures in 5 of these patients). The range of END mainly depended on T-status. In 16 patients, modified radical neck dissection (MRND) was performed. All other 50 patients underwent selective neck dissection (SND). The extensiveness of SND was the following: in 40 patients, levels I–III and V and in 10 patients levels II, III and V. Supplementary radiotherapy was carried out in 37 patients. Follow-up for the entire analysed group was at least 5 year long (between 5 and 24 years, mean 7.9 years). During follow-up, nine patients were diagnosed with locoregional relapse and ten patients were found to present distant metastases (lung, bones, liver and axillary lymph nodes). Twenty patients (30.3%) died due to the disease.

In search of risk factors for occult metastases to cervical lymph nodes, we analysed the following variables: age, sex, pT-Status (T1 + T2 vs. T3 + T4a), tumour size, skin invasion, facial nerve palsy, tumour fixation, extraparotid extension, localization (superficial lobe vs. whole gland), grade (high vs. intermediate/low), histology and intra/periparotid LN metastases. In all cases, histological revision was carried out according to the WHO classification of parotid gland carcinomas (2005) [1]. Due to the multitude of histological types, for statistical analysis, we divided them based on the classification by De Brito Santos et al., into three groups according to the risk of metastases to lymph nodes (low <20%, moderate 20–50% and high >50%). The first group comprised adenoid cystic carcinoma (AdCC) and mucoepidermoid carcinoma intermediate grade/low grade (MEC IG/LG) as well as papillary cystadenocarcinoma (PCA), the second comprised acinic cell carcinoma (AcCC), carcinoma ex pleomorphic adenoma (CXPA) and adenocarcinoma not other specified (AC NOS) LG, sebaceus carcinoma (SCa), and the third group comprised AC NOS high grade (HG), neuroendocrine (small cell) carcinoma (NCa), undifferentiated carcinoma (UC), MEC HG, squamous cell carcinoma (SCC) and salivary duct carcinoma (SDC) [7].

The relationships between the binary dependent variables and predictors of a categorical evaluated by calculating the odds ratio (OR) with 95% confidence interval and the Chi-square test. In the case of continuous predictor variables used logistic regression. Multivariate analysis was performed using logistic regression with the selection of variables stepwise backward. *p* values <0.05 were considered statistically significant. Statistical analysis was carried out using STATA 13.0 statistical package software (StataCorp, TX, USA).

A systematic review of original articles analysing the cervical nodal metastases in PPC was performed by searching electronic databases PUBMED and Scopus (January 1990 to December 2015) with the following keywords: parotid, salivary gland, carcinoma/cancer, elective, neck dissection, occult, lymph node metastasis and regional metastases. Searches were supplemented by scanning references of the articles included. For this analysis, we selected publications providing data on risk factors and locations of metastases to cervical lymph nodes in parotid gland carcinoma. To assess occult metastases to particular levels of cervical lymph nodes in a larger group of patients, we compiled the obtained data.

## Results

Occult metastases were observed in 20 out of all 66 patients (30.3%) who underwent END. In eight patients, the metastasis was single, four patients were diagnosed with metastases to two lymph nodes, three patients were diagnosed with metastases to three lymph nodes and four patients to four lymph nodes and there were isolated cases of metastases to five, six and ten lymph nodes. Metastases were most frequently observed in cases of AC NOS HG (5/11) and MEC HG (5/8), as well as CXPA (3/10). There were single cases of metastases being observed in MEC LG (1/6), AcCC LG (1/6), SDC (1/4), SCC (1/3), SCa (1/1), NCa (1/1), PCA (1/1). No metastases were observed in 10 AdCC and 5 UCa, as well as 2 AC NOS LG.

Out of all analysed variable, in a univariate analysis, statistical significance was found only for metastasis to intra/periparotid LN (*p* = 0.011), extraparotid extension (*p* = 0.019) and high risk histology (*p* = 0.023). They increased the risk of occult lymph node metastases by 6.1, 4.4 and 6.6 times, respectively. A multivariate analysis showed only first variable (*p* = 0.025) to be independent factor statistically significantly associated with presence of metastases in neck lymph nodes. Comparison of clinical-pathological data as potential risk factors of occult metastases in patients with cN0/pN− and cN0/pN+ is presented in Table 1.

**Table 1:** Comparison of clinical-pathological data of patients with cN0/pN− and cN0/pN+

	cN0/pN(+)	cN0/pN(−)	Univariate		Multivariate	
	20	46	OR	*p*	OR	*p*
Age (years)						
Min	11	15	1.01	0.599		ns
Max	82	90				
Average	62.1	59.6				
Sex						
Male	9	23	0.8	0.709		ns
Female	11	23				
pT-Status						
T1 + T2	8	22	1.2	0.793		ns
T3 + T4a	12	24				
Size (cm)						
Min	2	1	1.03	0.861		ns
Max	6	7				
Average (SD)	3.8 (±1.3)	3.6 (±1.5)				
Skin invasion	4	11	0.8	0.727		ns
Facial nerve palsy	5	9	1.4	0.620		ns
Fixation (tumour)	11	14	2.8	0.059	3.2	0.104
Extraparotid extension	7	5	4.4	0.019	4.3	0.053
Lobe						
Superficial	8	21	1.5	0.485		ns
Superficial and deep	12	25				
Grade						
High	16	30	2.1	0.230		ns
Intermediate/low	4	16				
Histology^a^						
High risk	15	17	6.6	0.023	2.6	0.172
Moderate risk	3	14	1.6	0.630		
Low risk	2	15	Reference group			
Intra/periparotid						
pN+	6	3	6.1	0.011	7.2	0.025
pN−	14	43				

*OR* odds ratio (95% confidence interval), *ns* no statistically

^a^High risk AC NOS high grade (HG), neuroendocrine (small cell) carcinoma (NCa), undifferentiated carcinoma (UC), MEC HG, squamous cell carcinoma (SCC), salivary duct carcinoma (SDC); moderate risk: acinic cell carcinoma (AcCC), carcinoma ex pleomorphic adenoma (CXPA), adenocarcinoma not other specified (AC NOS) LG, sebaceus carcinoma (SCa); low risk: adenoid cystic carcinoma (AdCC), mucoepidermoid carcinoma intermediate grade/low grade (MEC IG/LG), papillary cystadenocarcinoma (PCA)

The most common locations of occult metastases in the analysed group of 20 patients were level II 16/20 (80%), III 9/20 (45%) and V 6/20 (30%). Skip metastases were not observed. By compilation of our own material with data from the literature (5 series), we obtained a group of 80 patients with occult metastases to neck lymph nodes only, selected out of 650 patients with cN0 (12.3%). In this review, the proportion of metastases to particular levels was the following (presented in descending order): 69%—II, 22.5%—III, 20%—I, 16%—V and 7.5% in IV. Occurrence and localizations of occult LNM are presented in Table 2.

**Table 2:** Occurrence and localizations of occult cervical LNM

Series	pN+/cN0, *n* (%)	Neck level				
		I	II	III	IV	V
Present	20/66 (30.3%)	0	16	9	0	6
Shinomiya [10]	8/59 (13.5%)	1	7	0	0	0
Stenner [3]	3/58 (5.1%)	0	2	1	0	0
Kawata [11]	4/51 (7.8%)	0	4	1	0	0
Klussmann [5]	25/90 (31.2%)	12	18	0	0	6
Armstrong [12]	20/326 (6.1%)	3	8	7	6	1
Summary	80/650 (12.3%)	16 (20%)	55 (69%)	18 (22.5%)	6 (7.5%)	13 (16%)

Data refer only to the parotid gland, without intra/periparotid lymph nodes metastases

## Discussion

When discussing the problem of occult metastases, it is worth to take into consideration the staging of the neck to detect false cN0. Without doubt, clinical examination as the only method of neck assessment is not sufficient. It is necessary to apply imaging examinations (US, CT, MRI). Based on their study of 106 patients with SCC of oral cavity, Stuckensen et al. showed that ultrasound examination is one of the highest sensitivity—84%, while CT and MRI 66 and 64%, respectively [8]. Even though in the presented material, all patients underwent ultrasound examination and some of them also had CT or MRI, occult metastases were found in as many as 1/3 of patients identified as N0. Another problem pertains to exclusion of lymph node metastases, i.e. pN− based on their literature review, Ferlito et al. discussed the problem of actual pN− fin the context of pathological examination. They emphasised that ruling out micrometastasis is affected by the proportion of examined and removed lymph nodes, type of incision (layers), and use of IHC or other molecular techniques such as PCR or flow cytometry [9].

Occult metastases to cervical lymph nodes in PPC are diagnosed in 5.1–31.2% patients. In our analysis of 5 series, the result amounted to 12.3% (80 pN+ patients vs. 650 cN0) [3, 5, 10–12]. Based on the own material and literature (Table 3), it is obvious that the most important statistically significant risk factors for metastases to cervical lymph nodes are grade (7/10 series) and local stage: T (6/10) and its indicators, i.e. the size of the tumour (2/10), extraglandular infiltration (4/10) and nerve VII paresis (4/10) [2, 4, 5, 7, 10–14]. In the presented material, there was a high proportion of occult metastases observed (30%) and even though we did not observe statistical significance for T, in a uni- and multivariate analysis, we showed a significant effect of extraparotid extension, which in TNM classification appears in T3. In contrast, in a group of 58 patients with cT1/T2 parotid gland carcinoma analysed by Stenner et al., cervical occult metastases (without intra/periparotid) were found in only 5.1% of patients [3]. Among all presented series, only in the material by Lau et al., there was no statistical significance detected for variables associated with local stage [14]. Patients’ age turned out to be a significant clinical risk factor in three series and male sex in one series [2, 5, 13].

**Table 3:** The risk factors of cervical LNM

Factor series	Clinical/imaging						FNAB/FS/final histopathology		
	Age	Sex	T status	N.VII palsy	Size	Extraparotid extension	Grade	Histology	Other^a^
Present						U		U	
Shinomiya [10]			U	U			U		
Armstrong [12]			U		M		U/M	U	
Klussman [5]	U	U	U				U		
Stennert [4]			U						
De Brito Santos [7]			U/M	U		U		U/M	M
Bhattacharyya [2]	M			M	M	M	M	M	
Kawata [11]			U				U		
Frankenthaler [13]	M			U/M		U/M	M		U/M
Lau [14]							U	M	
Total	3/10	1/10	6/10	4/10	2/10	4/10	7/10	5/10	2/10

*FNAB* fine needle aspiration biopsy, *FS* frozen section, *U* univariate analysis, *M* multivariate analysis

^a^Other perilymphatic invasion and/or necrosis and/or desmoplasia

The problem of identification of histological risk factors of metastases to lymph nodes results from multitude of salivary gland carcinoma types. As pointed out in the literature, regional metastases are most common in SCC, AC NOS, MEC, CXPA, SDC, UCa and less common in AcCC and AdCC [2–4, 10, 11, 14, 15].

Based on literature review, Ferlito et al. emphasised that most primary SCC are incorrectly diagnosed MEC—a metastasis of skin cancer or squamous cell metaplasia, and AdCC does not give metastases via lymphatic path to lymph nodes, but it can involve them by continuity. Moreover, UC and Aca NOS are a kind of “umbrellas” used by pathologists to cover all types of carcinomas that they are not able to classify in a better way. In a summary of the results obtained by other authors, Ferlito et al. concluded that MEC HG, CXPA, UC, SDC, SCC and *adenosquamosum* (ASqC) tissues are an indication for elective neck dissection [9]. However, authors also point out to the fact that ASqCa is not included in the WHO classification of salivary glands and is identical with MEC HG. De Brito Santos et al. distinguished three groups of risk of cervical metastases (low <20%, moderate 20–50% and high >50%), according to histological features. The first group comprised AdCC and MEC IL/LG, the second AcCC, CXPA, MECa, and the third AC NOS, UC, MEC HG, SCC and SDC [7]. This distinction was used in our work and it seems to reflect well the relation between tissues and the prevalence of metastases to lymph nodes, even though in our material metastases did not occur in UC. Also, it should be remembered that histological groups of risk are associated with tumour grade. This distinction should be supplemented by all other histological types of salivary gland carcinomas. Final histopathological examinations in the work by De Brito Santos indicated that occult metastases were significantly affected by necrosis and/or desmoplasia within the tumour, as well as perineural infiltration; in the material by Frankenthaler et al., this list was supplemented with perilymphatic invasion [7, 13]. However, Zbären et al. emphasised that, with the use of fine needle aspiration biopsy (FNAB) and frozen section (FS), it is not possible to assess with 100% certainty the grade and histology of salivary gland tumour. This is why most of the information about these factors can only be obtained from the final histological examination of the excised tumour. Therefore, the decision to perform simultaneous END may not be made based on the histology and grade parameters only [16]. Naturally, this also pertains to other features that can only be assessed based on the histopathological examination, in particular issues associated with metastases to intra/periparotid lymph nodes (I/PLN).

Metastases to I/PLN seem to be a local factor (local feature of stage), not a regional one. I/PLN metastases are not included in any of the groups of neck lymph nodes. The *N* parameter in the TNM classification refers to regional, cervical lymph nodes, so I/PLN metastases should not be classified as occult metastases in patients with cN0 neck. This is why the previously presented data pertaining to the proportion of occult metastases are completely different. However, potential relation between metastases to I/PLN and occult neck metastases still remains a significant problem. Lim et al. showed that patients with cN0 neck and I/PLN metastases have statistically significant higher chance of locoregional recurrence and poorer prognosis [17]. In the published results from series of patients with cN0 salivary gland carcinomas, I/PLN metastases were coexistent with occult neck metastases (30–80%) [3, 5, 10, 12]. Some authors recommend total parotidectomy even in patients with early stage parotid gland carcinomas, since it is the only way to remove and identify all I/PLN metastases [3, 5]. In the presented material I/PLN metastases were diagnosed in 6/20 (30%) cN0/pN+ patients and 3/46 (6.5%) cN0/pN− patients. This difference was statistically significant both in the uni- and multivariate analysis and it seems to be another histopathological risk factor of occult metastases.

Without doubt, the most prevalent location of occult metastases was level II (approximately 70%), which should not be surprising, since the tail of the parotid gland lies anatomically in the neck [14]. I, III and V levels were involved in approximately 20% (±3%), while level IV was involved in only one series (<10%). Involvement of level V, especially Va, is not that obvious (3/6 series) and most probably it is associated with location and local stage of the tumour [3, 10–12, 15]. In the presented series and two other ones, there were no metastases in level I either [3, 11]. Teymoortash et al. pointed out to the fact that even though all the lymph is drained to the superficial and deep intersalivary lymph nodes, from the anteroinferior part of the parotid gland it may pass though the masseter directly to submandibular lymph nodes [18]. Skip metastases to cervical lymph nodes in cN0-3 parotid gland carcinoma occur in 18.5–33% of patients. They are most frequently observed in level III and/or IV [11, 12, 15]. Armstrong et al. reported that at removal of level II and III the risk of skip metastases being left is only 10% [12]. This is a significant conclusion especially that level IV was involved by occult metastases in only this one set of patients. Also, it should be remembered that skip metastases can result from imprecise identification of LN group during ND, e.g. on the line of the lower part of level III and upper part of level IV, which is identified based on the level of the cricoid cartilage or the cross of the omohyoid muscle with the internal jugular vein.

There are no unified indications for END and its scope for cases of PPC. Some authors believe that it should be performed in all cases of PPC, regardless of its stage and type [3, 11, 16]. Other authors recommend it only in local T3/T4 and/or high grade tumour and/or tumours of high risk histology [7, 10, 12]. Table 4 summarises all the indications and range of END according to literature reports.

**Table 4:** The indications and range of END according to the literature

Author	T-stage	Grade	END (neck level)				
Shinomiya [10]	T3, T4	High/low	I	II			
Stenner [3]	T1–T4	High/low		II	III		
Zbären [16]	T1–T4	High/low		II	III		
Armstrong [12]	T3–T4	High	I	II	III		
Kawata [11]	T1–T4	High/low	I	II	III		Va
Klussman [5]	T1–T4	High	Ib	II	III	IV	Va

## Conclusion

Based on the above-mentioned data, a conclusion can be drawn that END should definitely be performed in all T3/T4aN0 carcinomas with minimal range of level II and III. Removal of levels I and Va is recommended as well. In the case of T1/T2 carcinomas with high risk histology/high grade in FNAB, END should be performed including levels II and III.
